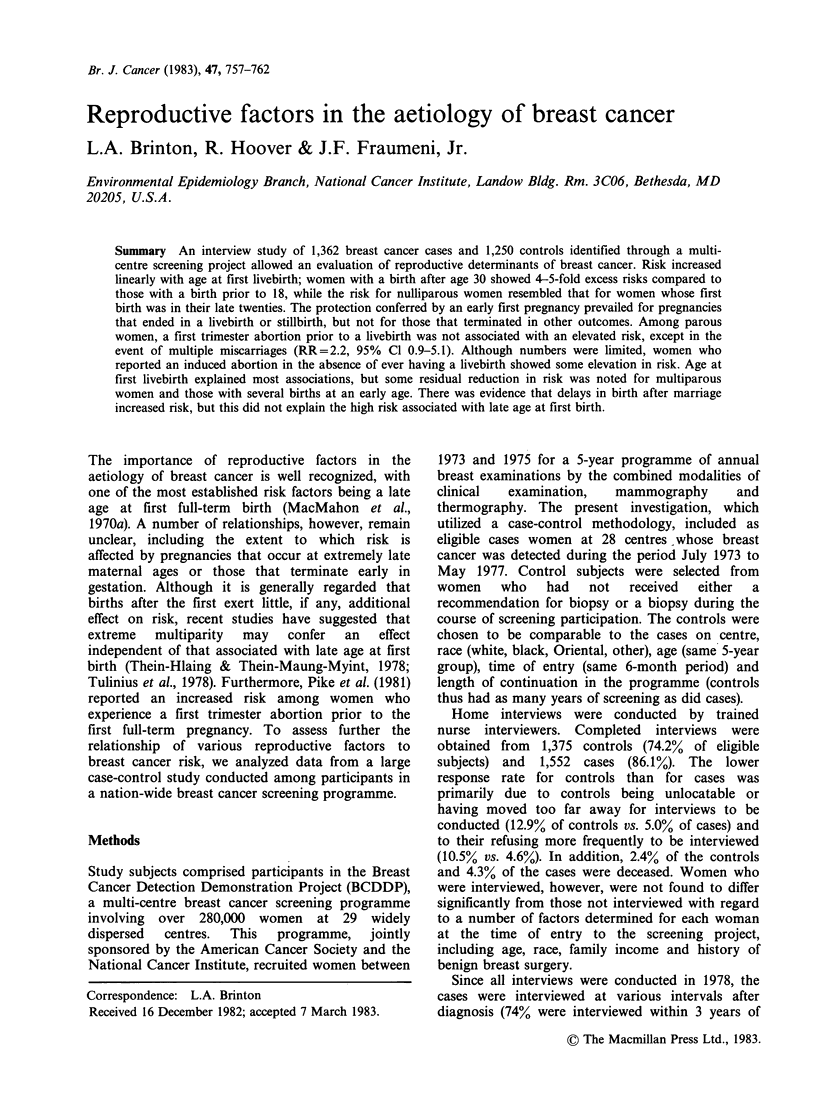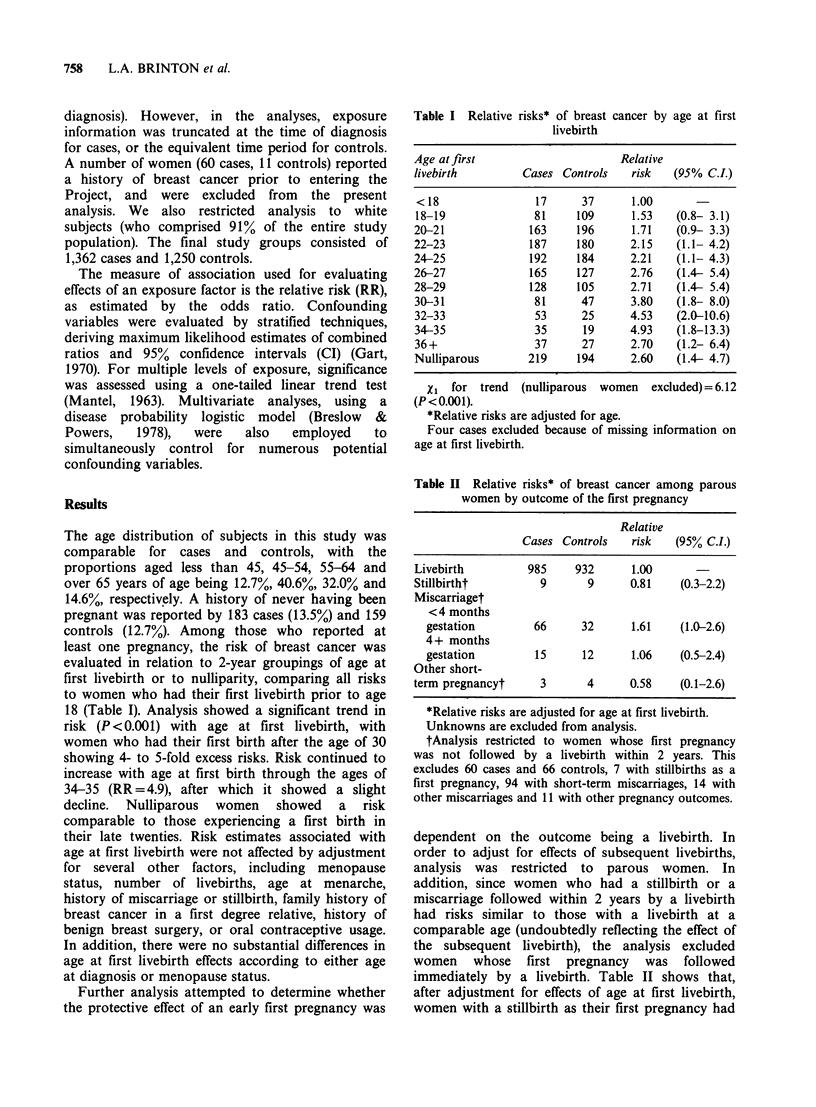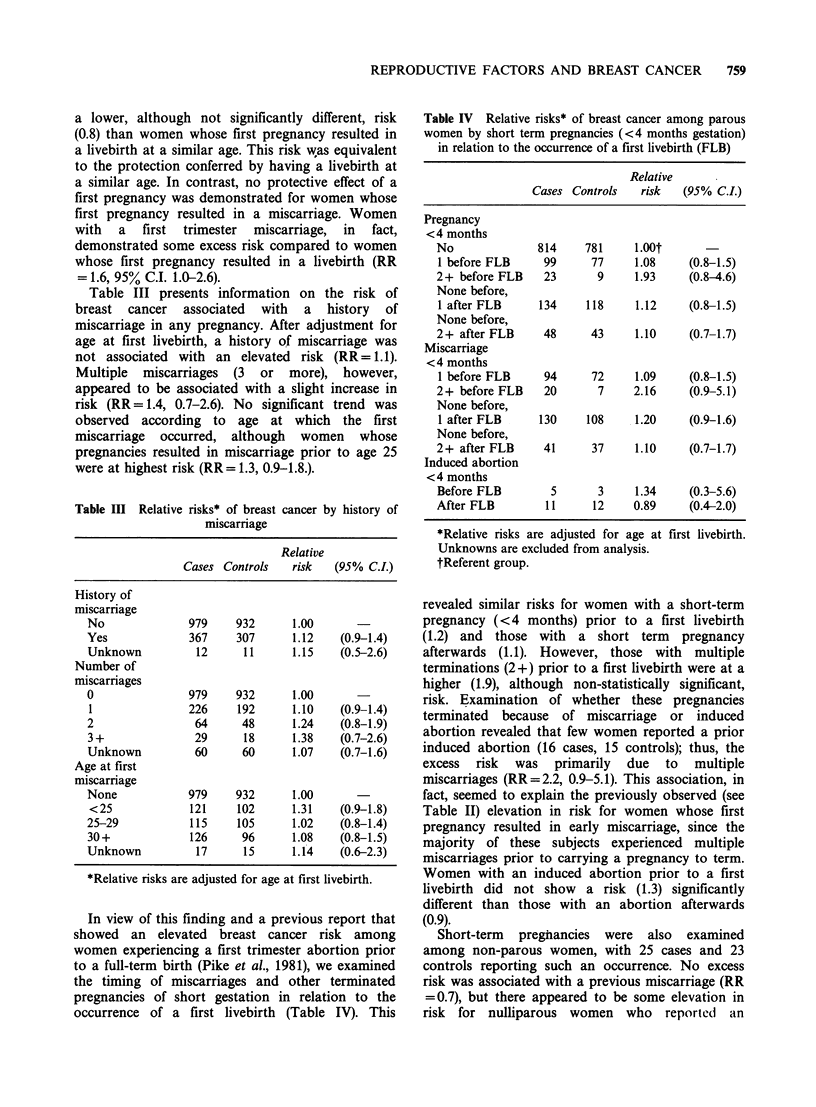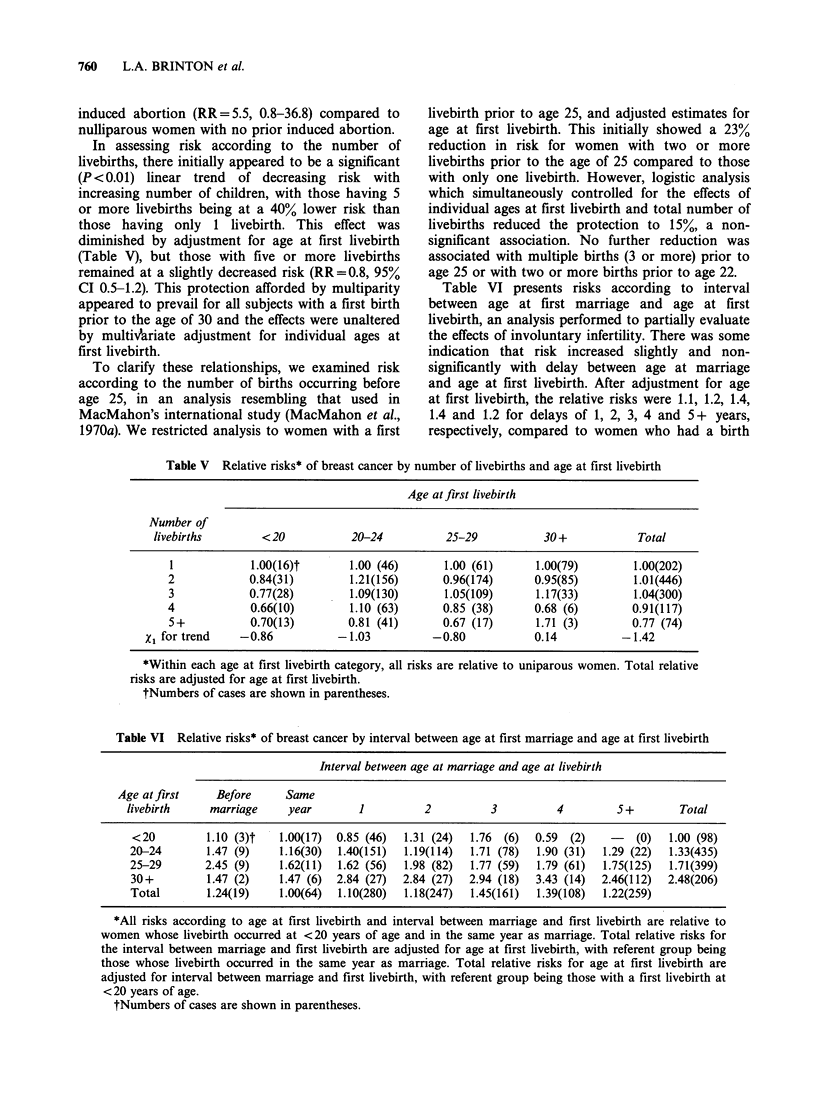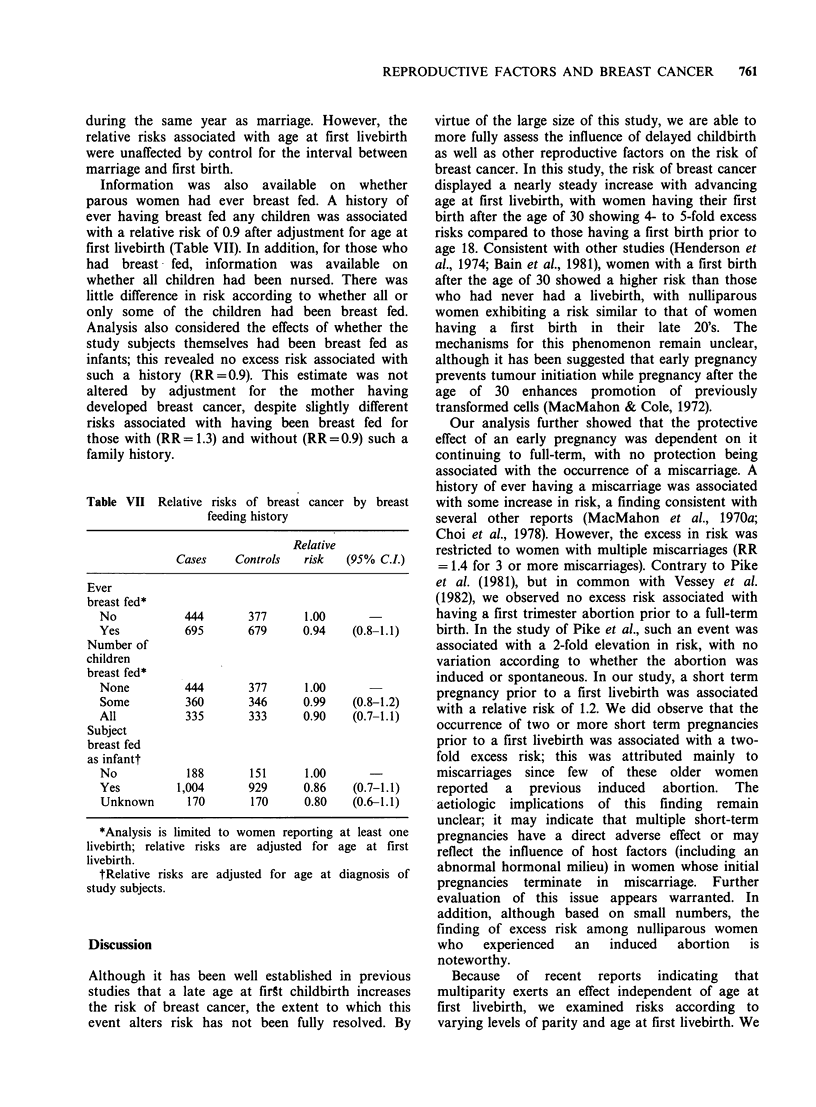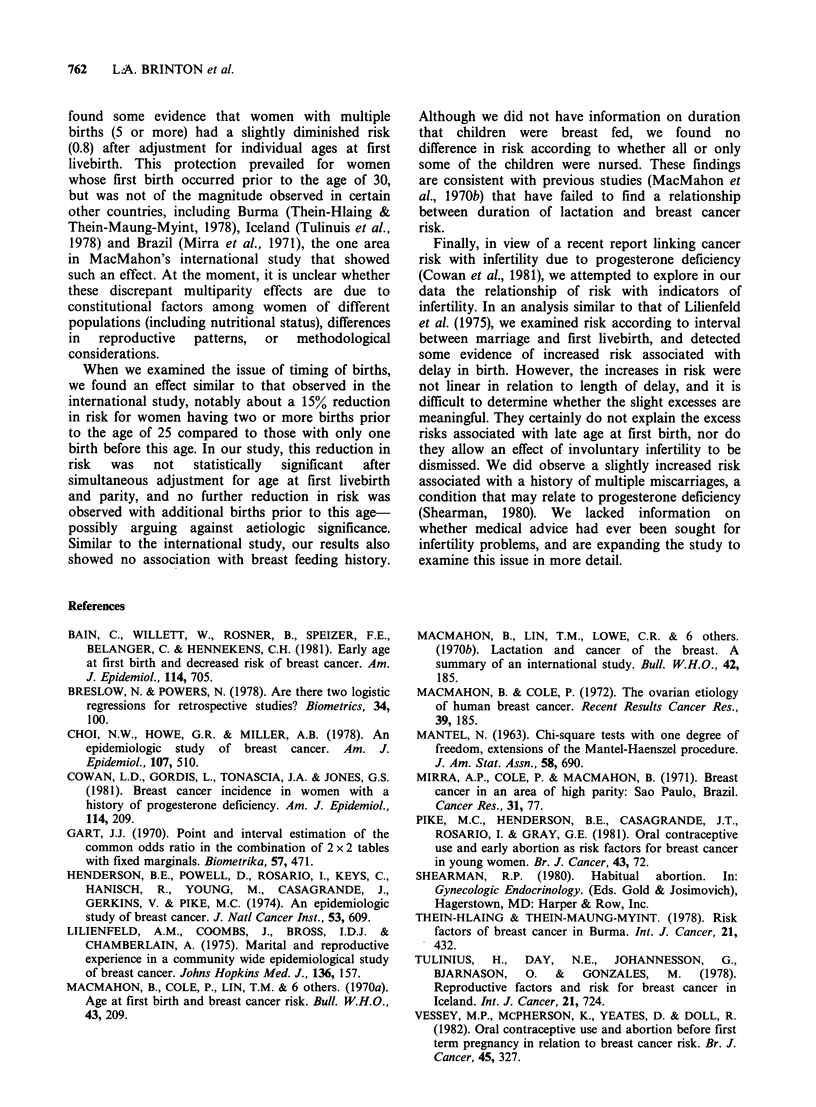# Reproductive factors in the aetiology of breast cancer.

**DOI:** 10.1038/bjc.1983.128

**Published:** 1983-06

**Authors:** L. A. Brinton, R. Hoover, J. F. Fraumeni

## Abstract

An interview study of 1,362 breast cancer cases and 1,250 controls identified through a multi-centre screening project allowed an evaluation of reproductive determinants of breast cancer. Risk increased linearly with age at first livebirth; women with a birth after age 30 showed 4-5-fold excess risks compared to those with a birth prior to 18, while the risk for nulliparous women resembled that for women whose first birth was in their late twenties. The protection conferred by an early first pregnancy prevailed for pregnancies that ended in a livebirth or stillbirth, but not for those that terminated in other outcomes. Among parous women, a first trimester abortion prior to a livebirth was not associated with an elevated risk, except in the event of multiple miscarriages (RR = 2.2, 95% Cl 0.9-5.1). Although numbers were limited, women who reported an induced abortion in the absence of ever having a livebirth showed some elevation in risk. Age at first livebirth explained most associations, but some residual reduction in risk was noted for multiparous women and those with several births at an early age. There was evidence that delays in birth after marriage increased risk, but this did not explain the high risk associated with late age at first birth.


*Br. J. Cancer* (1983), **47**, 757–762

### Reproductive factors in the aetiology of breast cancer

L.A. Brinton, R. Hoover & J.F. Fraumeni, Jr.

*Environmental Epidemiology Branch, National Cancer Institute, Landow Bldg. Rm. 3C06, Bethesda, MD 20205, U.S.A.*

**Summary** An interview study of 1,362 breast cancer cases and 1,250 controls identified through a multi-centre screening project allowed an evaluation of reproductive determinants of breast cancer. Risk increased linearly with age at first livebirth; women with a birth after age 30 showed 4–5-fold excess risks compared to those with a birth prior to 18, while the risk for nulliparous women resembled that for women whose first birth was in their late twenties. The protection conferred by an early first pregnancy prevailed for pregnancies that ended in a livebirth or stillbirth, but not for those that terminated in other outcomes. Among parous women, a first trimester abortion prior to a livebirth was not associated with an elevated risk, except in the event of multiple miscarriages (RR = 2.2, 95% Cl 0.9–5.1). Although numbers were limited, women who reported an induced abortion in the absence of ever having a livebirth showed some elevation in risk. Age at first livebirth explained most associations, but some residual reduction in risk was noted for multiparous women and those with several births at an early age. There was evidence that delays in birth after marriage increased risk, but this did not explain the high risk associated with late age at first birth.

The importance of reproductive factors in the aetiology of breast cancer is well recognized, with one of the most established risk factors being a late age at first full-term birth (MacMahon *et al.*, 1970*a*). A number of relationships, however, remain unclear, including the extent to which risk is affected by pregnancies that occur at extremely late maternal ages or those that terminate early in gestation. Although it is generally regarded that births after the first exert little, if any, additional effect on risk, recent studies have suggested that extreme multiparity may confer an effect independent of that associated with late age at first birth (Thein-Hlaing & Thein-Maung-Myint, 1978; Tulinius *et al.*, 1978). Furthermore, Pike *et al.* (1981) reported an increased risk among women who experience a first trimester abortion prior to the first full-term pregnancy. To assess further the relationship of various reproductive factors to breast cancer risk, we analyzed data from a large case-control study conducted among participants in a nation-wide breast cancer screening programme.

### Methods

Study subjects comprised participants in the Breast Cancer Detection Demonstration Project (BCDDP), a multi-centre breast cancer screening programme involving over 280,000 women at 29 widely dispersed centres. This programme, jointly sponsored by the American Cancer Society and the National Cancer Institute, recruited women between 1973 and 1975 for a 5-year programme of annual breast examinations by the combined modalities of clinical examination, mammography and thermography. The present investigation, which utilized a case-control methodology, included as eligible cases women at 28 centres whose breast cancer was detected during the period July 1973 to May 1977. Control subjects were selected from women who had not received either a recommendation for biopsy or a biopsy during the course of screening participation. The controls were chosen to be comparable to the cases on centre, race (white, black, Oriental, other), age (same 5-year group), time of entry (same 6-month period) and length of continuation in the programme (controls thus had as many years of screening as did cases).

Home interviews were conducted by trained nurse interviewers. Completed interviews were obtained from 1,375 controls (74.2% of eligible subjects) and 1,552 cases (86.1%). The lower response rate for controls than for cases was primarily due to controls being unlocatable or having moved too far away for interviews to be conducted (12.9% of controls *vs.* 5.0% of cases) and to their refusing more frequently to be interviewed (10.5% *vs.* 4.6%). In addition, 2.4% of the controls and 4.3% of the cases were deceased. Women who were interviewed, however, were not found to differ significantly from those not interviewed with regard to a number of factors determined for each woman at the time of entry to the screening project, including age, race, family income and history of benign breast surgery.

Since all interviews were conducted in 1978, the cases were interviewed at various intervals after diagnosis (74% were interviewed within 3 years of

Correspondence: L.A. Brinton
Received 16 December 1982; accepted 7 March 1983.

© The Macmillan Press Ltd., 1983.

diagnosis). However, in the analyses, exposure information was truncated at the time of diagnosis for cases, or the equivalent time period for controls. A number of women (60 cases, 11 controls) reported a history of breast cancer prior to entering the Project, and were excluded from the present analysis. We also restricted analysis to white subjects (who comprised 91% of the entire study population). The final study groups consisted of 1,362 cases and 1,250 controls.

The measure of association used for evaluating effects of an exposure factor is the relative risk (RR), as estimated by the odds ratio. Confounding variables were evaluated by stratified techniques, deriving maximum likelihood estimates of combined ratios and 95% confidence intervals (CI) (Gart, 1970). For multiple levels of exposure, significance was assessed using a one-tailed linear trend test (Mantel, 1963). Multivariate analyses, using a disease probability logistic model (Breslow & Powers, 1978), were also employed to simultaneously control for numerous potential confounding variables.

### Results

The age distribution of subjects in this study was comparable for cases and controls, with the proportions aged less than 45, 45–54, 55–64 and over 65 years of age being 12.7%, 40.6%, 32.0% and 14.6%, respectively. A history of never having been pregnant was reported by 183 cases (13.5%) and 159 controls (12.7%). Among those who reported at least one pregnancy, the risk of breast cancer was evaluated in relation to 2-year groupings of age at first livebirth or to nulliparity, comparing all risks to women who had their first livebirth prior to age 18 (Table I). Analysis showed a significant trend in risk ($P<0.001$) with age at first livebirth, with women who had their first birth after the age of 30 showing 4- to 5-fold excess risks. Risk continued to increase with age at first birth through the ages of 34–35 (RR = 4.9), after which it showed a slight decline. Nulliparous women showed a risk comparable to those experiencing a first birth in their late twenties. Risk estimates associated with age at first livebirth were not affected by adjustment for several other factors, including menopause status, number of livebirths, age at menarche, history of miscarriage or stillbirth, family history of breast cancer in a first degree relative, history of benign breast surgery, or oral contraceptive usage. In addition, there were no substantial differences in age at first livebirth effects according to either age at diagnosis or menopause status.

Further analysis attempted to determine whether the protective effect of an early first pregnancy was dependent on the outcome being a livebirth. In order to adjust for effects of subsequent livebirths, analysis was restricted to parous women. In addition, since women who had a stillbirth or a miscarriage followed within 2 years by a livebirth had risks similar to those with a livebirth at a comparable age (undoubtedly reflecting the effect of the subsequent livebirth), the analysis excluded women whose first pregnancy was followed immediately by a livebirth. Table II shows that, after adjustment for effects of age at first livebirth, women with a stillbirth as their first pregnancy had

**Table I** Relative risks* of breast cancer by age at first livebirth

| *Age at first livebirth* | *Cases* | *Controls* | *Relative risk* | *(95% C.I.)* |
|---|---|---|---|---|
| <18 | 17 | 37 | 1.00 | — |
| 18–19 | 81 | 109 | 1.53 | (0.8– 3.1) |
| 20–21 | 163 | 196 | 1.71 | (0.9– 3.3) |
| 22–23 | 187 | 180 | 2.15 | (1.1– 4.2) |
| 24–25 | 192 | 184 | 2.21 | (1.1– 4.3) |
| 26–27 | 165 | 127 | 2.76 | (1.4– 5.4) |
| 28–29 | 128 | 105 | 2.71 | (1.4– 5.4) |
| 30–31 | 81 | 47 | 3.80 | (1.8– 8.0) |
| 32–33 | 53 | 25 | 4.53 | (2.0–10.6) |
| 34–35 | 35 | 19 | 4.93 | (1.8–13.3) |
| 36+ | 37 | 27 | 2.70 | (1.2– 6.4) |
| Nulliparous | 219 | 194 | 2.60 | (1.4– 4.7) |

$\chi_1$ for trend (nulliparous women excluded) = 6.12 ($P<0.001$).

*Relative risks are adjusted for age.

Four cases excluded because of missing information on age at first livebirth.

**Table II** Relative risks* of breast cancer among parous women by outcome of the first pregnancy

| | *Cases* | *Controls* | *Relative risk* | *(95% C.I.)* |
|---|---|---|---|---|
| Livebirth | 985 | 932 | 1.00 | — |
| Stillbirth† | 9 | 9 | 0.81 | (0.3–2.2) |
| Miscarriage† | | | | |
| <4 months gestation | 66 | 32 | 1.61 | (1.0–2.6) |
| 4+ months gestation | 15 | 12 | 1.06 | (0.5–2.4) |
| Other short-term pregnancy† | 3 | 4 | 0.58 | (0.1–2.6) |

*Relative risks are adjusted for age at first livebirth.

Unknowns are excluded from analysis.

†Analysis restricted to women whose first pregnancy was not followed by a livebirth within 2 years. This excludes 60 cases and 66 controls, 7 with stillbirths as a first pregnancy, 94 with short-term miscarriages, 14 with other miscarriages and 11 with other pregnancy outcomes.

a lower, although not significantly different, risk (0.8) than women whose first pregnancy resulted in a livebirth at a similar age. This risk was equivalent to the protection conferred by having a livebirth at a similar age. In contrast, no protective effect of a first pregnancy was demonstrated for women whose first pregnancy resulted in a miscarriage. Women with a first trimester miscarriage, in fact, demonstrated some excess risk compared to women whose first pregnancy resulted in a livebirth (RR = 1.6, 95% C.I. 1.0–2.6).

Table III presents information on the risk of breast cancer associated with a history of miscarriage in any pregnancy. After adjustment for age at first livebirth, a history of miscarriage was not associated with an elevated risk (RR = 1.1). Multiple miscarriages (3 or more), however, appeared to be associated with a slight increase in risk (RR = 1.4, 0.7–2.6). No significant trend was observed according to age at which the first miscarriage occurred, although women whose pregnancies resulted in miscarriage prior to age 25 were at highest risk (RR = 1.3, 0.9–1.8.).

**Table III** Relative risks* of breast cancer by history of miscarriage

| | *Cases* | *Controls* | *Relative risk* | *(95% C.I.)* |
|---|---|---|---|---|
| History of miscarriage | | | | |
| No | 979 | 932 | 1.00 | — |
| Yes | 367 | 307 | 1.12 | (0.9–1.4) |
| Unknown | 12 | 11 | 1.15 | (0.5–2.6) |
| Number of miscarriages | | | | |
| 0 | 979 | 932 | 1.00 | — |
| 1 | 226 | 192 | 1.10 | (0.9–1.4) |
| 2 | 64 | 48 | 1.24 | (0.8–1.9) |
| 3+ | 29 | 18 | 1.38 | (0.7–2.6) |
| Unknown | 60 | 60 | 1.07 | (0.7–1.6) |
| Age at first miscarriage | | | | |
| None | 979 | 932 | 1.00 | — |
| <25 | 121 | 102 | 1.31 | (0.9–1.8) |
| 25–29 | 115 | 105 | 1.02 | (0.8–1.4) |
| 30+ | 126 | 96 | 1.08 | (0.8–1.5) |
| Unknown | 17 | 15 | 1.14 | (0.6–2.3) |

*Relative risks are adjusted for age at first livebirth.

In view of this finding and a previous report that showed an elevated breast cancer risk among women experiencing a first trimester abortion prior to a full-term birth (Pike *et al.*, 1981), we examined the timing of miscarriages and other terminated pregnancies of short gestation in relation to the occurrence of a first livebirth (Table IV). This revealed similar risks for women with a short-term pregnancy (<4 months) prior to a first livebirth (1.2) and those with a short term pregnancy afterwards (1.1). However, those with multiple terminations (2+) prior to a first livebirth were at a higher (1.9), although non-statistically significant, risk. Examination of whether these pregnancies terminated because of miscarriage or induced abortion revealed that few women reported a prior induced abortion (16 cases, 15 controls); thus, the excess risk was primarily due to multiple miscarriages (RR = 2.2, 0.9–5.1). This association, in fact, seemed to explain the previously observed (see Table II) elevation in risk for women whose first pregnancy resulted in early miscarriage, since the majority of these subjects experienced multiple miscarriages prior to carrying a pregnancy to term. Women with an induced abortion prior to a first livebirth did not show a risk (1.3) significantly different than those with an abortion afterwards (0.9).

**Table IV** Relative risks* of breast cancer among parous women by short term pregnancies (<4 months gestation) in relation to the occurrence of a first livebirth (FLB)

| | *Cases* | *Controls* | *Relative risk* | *(95% C.I.)* |
|---|---|---|---|---|
| Pregnancy <4 months | | | | |
| No | 814 | 781 | 1.00† | — |
| 1 before FLB | 99 | 77 | 1.08 | (0.8–1.5) |
| 2+ before FLB | 23 | 9 | 1.93 | (0.8–4.6) |
| None before, 1 after FLB | 134 | 118 | 1.12 | (0.8–1.5) |
| None before, 2+ after FLB | 48 | 43 | 1.10 | (0.7–1.7) |
| Miscarriage <4 months | | | | |
| 1 before FLB | 94 | 72 | 1.09 | (0.8–1.5) |
| 2+ before FLB | 20 | 7 | 2.16 | (0.9–5.1) |
| None before, 1 after FLB | 130 | 108 | 1.20 | (0.9–1.6) |
| None before, 2+ after FLB | 41 | 37 | 1.10 | (0.7–1.7) |
| Induced abortion <4 months | | | | |
| Before FLB | 5 | 3 | 1.34 | (0.3–5.6) |
| After FLB | 11 | 12 | 0.89 | (0.4–2.0) |

*Relative risks are adjusted for age at first livebirth. Unknowns are excluded from analysis.
†Referent group.

Short-term pregnancies were also examined among non-parous women, with 25 cases and 23 controls reporting such an occurrence. No excess risk was associated with a previous miscarriage (RR = 0.7), but there appeared to be some elevation in risk for nulliparous women who reported an induced abortion (RR = 5.5, 0.8–36.8) compared to nulliparous women with no prior induced abortion.

In assessing risk according to the number of livebirths, there initially appeared to be a significant ($P < 0.01$) linear trend of decreasing risk with increasing number of children, with those having 5 or more livebirths being at a 40% lower risk than those having only 1 livebirth. This effect was diminished by adjustment for age at first livebirth (Table V), but those with five or more livebirths remained at a slightly decreased risk (RR = 0.8, 95% CI 0.5–1.2). This protection afforded by multiparity appeared to prevail for all subjects with a first birth prior to the age of 30 and the effects were unaltered by multivariate adjustment for individual ages at first livebirth.

To clarify these relationships, we examined risk according to the number of births occurring before age 25, in an analysis resembling that used in MacMahon's international study (MacMahon et al., 1970a). We restricted analysis to women with a first livebirth prior to age 25, and adjusted estimates for age at first livebirth. This initially showed a 23% reduction in risk for women with two or more livebirths prior to the age of 25 compared to those with only one livebirth. However, logistic analysis which simultaneously controlled for the effects of individual ages at first livebirth and total number of livebirths reduced the protection to 15%, a non-significant association. No further reduction was associated with multiple births (3 or more) prior to age 25 or with two or more births prior to age 22.

Table VI presents risks according to interval between age at first marriage and age at first livebirth, an analysis performed to partially evaluate the effects of involuntary infertility. There was some indication that risk increased slightly and non-significantly with delay between age at marriage and age at first livebirth. After adjustment for age at first livebirth, the relative risks were 1.1, 1.2, 1.4, 1.4 and 1.2 for delays of 1, 2, 3, 4 and 5+ years, respectively, compared to women who had a birth

**Table V** Relative risks* of breast cancer by number of livebirths and age at first livebirth

| *Number of livebirths* | *Age at first livebirth* <20 | 20–24 | 25–29 | 30+ | *Total* |
|---|---|---|---|---|---|
| 1 | 1.00(16)† | 1.00 (46) | 1.00 (61) | 1.00(79) | 1.00(202) |
| 2 | 0.84(31) | 1.21(156) | 0.96(174) | 0.95(85) | 1.01(446) |
| 3 | 0.77(28) | 1.09(130) | 1.05(109) | 1.17(33) | 1.04(300) |
| 4 | 0.66(10) | 1.10 (63) | 0.85 (38) | 0.68 (6) | 0.91(117) |
| 5+ | 0.70(13) | 0.81 (41) | 0.67 (17) | 1.71 (3) | 0.77 (74) |
| $\chi_1$ for trend | −0.86 | −1.03 | −0.80 | 0.14 | −1.42 |

*Within each age at first livebirth category, all risks are relative to uniparous women. Total relative risks are adjusted for age at first livebirth.

†Numbers of cases are shown in parentheses.

**Table VI** Relative risks* of breast cancer by interval between age at first marriage and age at first livebirth

| *Age at first livebirth* | *Interval between age at marriage and age at livebirth* Before marriage | Same year | 1 | 2 | 3 | 4 | 5+ | *Total* |
|---|---|---|---|---|---|---|---|---|
| <20 | 1.10 (3)† | 1.00(17) | 0.85 (46) | 1.31 (24) | 1.76 (6) | 0.59 (2) | — (0) | 1.00 (98) |
| 20–24 | 1.47 (9) | 1.16(30) | 1.40(151) | 1.19(114) | 1.71 (78) | 1.90 (31) | 1.29 (22) | 1.33(435) |
| 25–29 | 2.45 (9) | 1.62(11) | 1.62 (56) | 1.98 (82) | 1.77 (59) | 1.79 (61) | 1.75(125) | 1.71(399) |
| 30+ | 1.47 (2) | 1.47 (6) | 2.84 (27) | 2.84 (27) | 2.94 (18) | 3.43 (14) | 2.46(112) | 2.48(206) |
| Total | 1.24(19) | 1.00(64) | 1.10(280) | 1.18(247) | 1.45(161) | 1.39(108) | 1.22(259) | |

*All risks according to age at first livebirth and interval between marriage and first livebirth are relative to women whose livebirth occurred at <20 years of age and in the same year as marriage. Total relative risks for the interval between marriage and first livebirth are adjusted for age at first livebirth, with referent group being those whose livebirth occurred in the same year as marriage. Total relative risks for age at first livebirth are adjusted for interval between marriage and first livebirth, with referent group being those with a first livebirth at <20 years of age.

†Numbers of cases are shown in parentheses.

during the same year as marriage. However, the relative risks associated with age at first livebirth were unaffected by control for the interval between marriage and first birth.

Information was also available on whether parous women had ever breast fed. A history of ever having breast fed any children was associated with a relative risk of 0.9 after adjustment for age at first livebirth (Table VII). In addition, for those who had breast fed, information was available on whether all children had been nursed. There was little difference in risk according to whether all or only some of the children had been breast fed. Analysis also considered the effects of whether the study subjects themselves had been breast fed as infants; this revealed no excess risk associated with such a history (RR=0.9). This estimate was not altered by adjustment for the mother having developed breast cancer, despite slightly different risks associated with having been breast fed for those with (RR=1.3) and without (RR=0.9) such a family history.

**Table VII** Relative risks of breast cancer by breast feeding history

| | *Cases* | *Controls* | *Relative risk* | *(95% C.I.)* |
|---|---|---|---|---|
| Ever breast fed* | | | | |
| No | 444 | 377 | 1.00 | — |
| Yes | 695 | 679 | 0.94 | (0.8–1.1) |
| Number of children breast fed* | | | | |
| None | 444 | 377 | 1.00 | — |
| Some | 360 | 346 | 0.99 | (0.8–1.2) |
| All | 335 | 333 | 0.90 | (0.7–1.1) |
| Subject breast fed as infant† | | | | |
| No | 188 | 151 | 1.00 | — |
| Yes | 1,004 | 929 | 0.86 | (0.7–1.1) |
| Unknown | 170 | 170 | 0.80 | (0.6–1.1) |

*Analysis is limited to women reporting at least one livebirth; relative risks are adjusted for age at first livebirth.

†Relative risks are adjusted for age at diagnosis of study subjects.

### Discussion

Although it has been well established in previous studies that a late age at first childbirth increases the risk of breast cancer, the extent to which this event alters risk has not been fully resolved. By virtue of the large size of this study, we are able to more fully assess the influence of delayed childbirth as well as other reproductive factors on the risk of breast cancer. In this study, the risk of breast cancer displayed a nearly steady increase with advancing age at first livebirth, with women having their first birth after the age of 30 showing 4- to 5-fold excess risks compared to those having a first birth prior to age 18. Consistent with other studies (Henderson *et al.*, 1974; Bain *et al.*, 1981), women with a first birth after the age of 30 showed a higher risk than those who had never had a livebirth, with nulliparous women exhibiting a risk similar to that of women having a first birth in their late 20's. The mechanisms for this phenomenon remain unclear, although it has been suggested that early pregnancy prevents tumour initiation while pregnancy after the age of 30 enhances promotion of previously transformed cells (MacMahon & Cole, 1972).

Our analysis further showed that the protective effect of an early pregnancy was dependent on it continuing to full-term, with no protection being associated with the occurrence of a miscarriage. A history of ever having a miscarriage was associated with some increase in risk, a finding consistent with several other reports (MacMahon *et al.*, 1970*a*; Choi *et al.*, 1978). However, the excess in risk was restricted to women with multiple miscarriages (RR =1.4 for 3 or more miscarriages). Contrary to Pike *et al.* (1981), but in common with Vessey *et al.* (1982), we observed no excess risk associated with having a first trimester abortion prior to a full-term birth. In the study of Pike *et al.*, such an event was associated with a 2-fold elevation in risk, with no variation according to whether the abortion was induced or spontaneous. In our study, a short term pregnancy prior to a first livebirth was associated with a relative risk of 1.2. We did observe that the occurrence of two or more short term pregnancies prior to a first livebirth was associated with a two-fold excess risk; this was attributed mainly to miscarriages since few of these older women reported a previous induced abortion. The aetiologic implications of this finding remain unclear; it may indicate that multiple short-term pregnancies have a direct adverse effect or may reflect the influence of host factors (including an abnormal hormonal milieu) in women whose initial pregnancies terminate in miscarriage. Further evaluation of this issue appears warranted. In addition, although based on small numbers, the finding of excess risk among nulliparous women who experienced an induced abortion is noteworthy.

Because of recent reports indicating that multiparity exerts an effect independent of age at first livebirth, we examined risks according to varying levels of parity and age at first livebirth. We found some evidence that women with multiple births (5 or more) had a slightly diminished risk (0.8) after adjustment for individual ages at first livebirth. This protection prevailed for women whose first birth occurred prior to the age of 30, but was not of the magnitude observed in certain other countries, including Burma (Thein-Hlaing & Thein-Maung-Myint, 1978), Iceland (Tulinuis *et al.*, 1978) and Brazil (Mirra *et al.*, 1971), the one area in MacMahon's international study that showed such an effect. At the moment, it is unclear whether these discrepant multiparity effects are due to constitutional factors among women of different populations (including nutritional status), differences in reproductive patterns, or methodological considerations.

When we examined the issue of timing of births, we found an effect similar to that observed in the international study, notably about a 15% reduction in risk for women having two or more births prior to the age of 25 compared to those with only one birth before this age. In our study, this reduction in risk was not statistically significant after simultaneous adjustment for age at first livebirth and parity, and no further reduction in risk was observed with additional births prior to this age—possibly arguing against aetiologic significance. Similar to the international study, our results also showed no association with breast feeding history. Although we did not have information on duration that children were breast fed, we found no difference in risk according to whether all or only some of the children were nursed. These findings are consistent with previous studies (MacMahon *et al.*, 1970*b*) that have failed to find a relationship between duration of lactation and breast cancer risk.

Finally, in view of a recent report linking cancer risk with infertility due to progesterone deficiency (Cowan *et al.*, 1981), we attempted to explore in our data the relationship of risk with indicators of infertility. In an analysis similar to that of Lilienfeld *et al.* (1975), we examined risk according to interval between marriage and first livebirth, and detected some evidence of increased risk associated with delay in birth. However, the increases in risk were not linear in relation to length of delay, and it is difficult to determine whether the slight excesses are meaningful. They certainly do not explain the excess risks associated with late age at first birth, nor do they allow an effect of involuntary infertility to be dismissed. We did observe a slightly increased risk associated with a history of multiple miscarriages, a condition that may relate to progesterone deficiency (Shearman, 1980). We lacked information on whether medical advice had ever been sought for infertility problems, and are expanding the study to examine this issue in more detail.

### References

BAIN, C., WILLETT, W., ROSNER, B., SPEIZER, F.E., BELANGER, C. & HENNEKENS, C.H. (1981). Early age at first birth and decreased risk of breast cancer. *Am. J. Epidemiol.*, **114**, 705.

BRESLOW, N. & POWERS, N. (1978). Are there two logistic regressions for retrospective studies? *Biometrics*, **34**, 100.

CHOI, N.W., HOWE, G.R. & MILLER, A.B. (1978). An epidemiologic study of breast cancer. *Am. J. Epidemiol.*, **107**, 510.

COWAN, L.D., GORDIS, L., TONASCIA, J.A. & JONES, G.S. (1981). Breast cancer incidence in women with a history of progesterone deficiency. *Am. J. Epidemiol.*, **114**, 209.

GART, J.J. (1970). Point and interval estimation of the common odds ratio in the combination of 2 × 2 tables with fixed marginals. *Biometrika*, **57**, 471.

HENDERSON, B.E., POWELL, D., ROSARIO, I., KEYS, C., HANISCH, R., YOUNG, M., CASAGRANDE, J., GERKINS, V. & PIKE, M.C. (1974). An epidemiologic study of breast cancer. *J. Natl Cancer Inst.*, **53**, 609.

LILIENFELD, A.M., COOMBS, J., BROSS, I.D.J. & CHAMBERLAIN, A. (1975). Marital and reproductive experience in a community wide epidemiological study of breast cancer. *Johns Hopkins Med. J.*, **136**, 157.

MACMAHON, B., COLE, P., LIN, T.M. & 6 others. (1970*a*). Age at first birth and breast cancer risk. *Bull. W.H.O.*, **43**, 209.

MACMAHON, B., LIN, T.M., LOWE, C.R. & 6 others. (1970*b*). Lactation and cancer of the breast. A summary of an international study. *Bull. W.H.O.*, **42**, 185.

MACMAHON, B. & COLE, P. (1972). The ovarian etiology of human breast cancer. *Recent Results Cancer Res.*, **39**, 185.

MANTEL, N. (1963). Chi-square tests with one degree of freedom, extensions of the Mantel-Haenszel procedure. *J. Am. Stat. Assn.*, **58**, 690.

MIRRA, A.P., COLE, P. & MACMAHON, B. (1971). Breast cancer in an area of high parity: Sao Paulo, Brazil. *Cancer Res.*, **31**, 77.

PIKE, M.C., HENDERSON, B.E., CASAGRANDE, J.T., ROSARIO, I. & GRAY, G.E. (1981). Oral contraceptive use and early abortion as risk factors for breast cancer in young women. *Br. J. Cancer*, **43**, 72.

SHEARMAN, R.P. (1980). Habitual abortion. In: *Gynecologic Endocrinology*. (Eds. Gold & Josimovich), Hagerstown, MD: Harper & Row, Inc.

THEIN-HLAING & THEIN-MAUNG-MYINT. (1978). Risk factors of breast cancer in Burma. *Int. J. Cancer*, **21**, 432.

TULINIUS, H., DAY, N.E., JOHANNESSON, G., BJARNASON, O. & GONZALES, M. (1978). Reproductive factors and risk for breast cancer in Iceland. *Int. J. Cancer*, **21**, 724.

VESSEY, M.P., MCPHERSON, K., YEATES, D. & DOLL, R. (1982). Oral contraceptive use and abortion before first term pregnancy in relation to breast cancer risk. *Br. J. Cancer*, **45**, 327.